# Tissue-Specific Distribution of Ginsenosides in Different Aged Ginseng and Antioxidant Activity of Ginseng Leaf

**DOI:** 10.3390/molecules191117381

**Published:** 2014-10-28

**Authors:** Ying-Chun Zhang, Geng Li, Chao Jiang, Bin Yang, Hong-Jun Yang, Hai-Yu Xu, Lu-Qi Huang

**Affiliations:** 1 State Key Laboratory Breeding Base of Dao-di Herbs, National Resource Center for Chinese Materia Medica, China Academy of Chinese Medical Sciences, Beijing 100700, China; E-Mail: yczhang1203@126.com; 2 Institute of Natural Medicine and Chinese Medicine Resources, Beijing Normal University, Beijing 100700, China; E-Mails: 13810507641@163.com (G.L.); jiangchao0411@126.com (C.J.); 3 National Integrative Medicine Center for Cardiovascular Disease, China-Japan Friendship Hospital, Beijing 100700, China; 4 Institute of Chinese Materia Medica, China Academy of Chinese Medical Sciences, Beijing 100700, China; E-Mails: ybinmm@hotmail.com (B.Y.); hongjun0420@vip.sina.com (H.-J.Y.); hyxu627@126.com (H.-Y.X.)

**Keywords:** ginseng, ginsenosides, UPLC, quantitative analysis, antioxidant activity

## Abstract

The aim of this study was to systematically evaluate the effect of the cultivation year on the quality of different ginseng tissues. Qualitative and quantitative analyses of ginsenosides were conducted using a UPLC-UV-MS method. Eight main ginsenosides in three tissues (leaf, rhizome and main root) and four parts (periderm, phloem, cambium and xylem) of ginseng aged from 1 to 13 years were determined using a UPLC-PDA method. Additionally, the antioxidant capacities of ginseng leaves were analyzed by the DPPH, ABTS and HRSA methods. It was found that the contents of ginsenosides increased with cultivation years, causing a sequential content change of ginsenosides in an organ-specific manner: leaf > rhizome > main root. The ratio between protopanaxatriol (PPT, Rg_1_, Re and RF) and protopanaxadiol (PPD, Rb_1_, Rb_2_, RC and Rd) in the main root remained stable (about 1.0), while it increased in leaf from 1.37 to 3.14 and decreased in the rhizome from 0.99 to 0.72. The amount of ginsenosides accumulated in the periderm was 45.48 mg/g, which was more than twice as high compared with the other three parts. Furthermore, the antioxidant activities of ginseng leaves were measured as Trolox equivalents, showing that antioxidant activity increased along with time of cultivation. The results show that the best harvest time for shizhu ginseng is the fifth year of cultivation, and the root and rhizome could be used together within seven planting years for their similar PPT/PPD level. Besides, the quality of the ginseng products would be enhanced with the periderm. The ginseng leaf is rich in ginsenosides and has potential application for its antioxidant capacity.

## 1. Introduction

Ginseng, the root and rhizome of *Panax ginseng* C. A. Mey., is a well-known and precious traditional Chinese medicine, which has been widely used in the clinic for several thousand years due to its medical value for curing central nervous system disease, cardiovascular system disease, endocrine system disease, cancer and so on [1,2,3,4]. According to the large amount of previous investigations on ginseng, the pharmacological activities of ginseng are mainly attributed to the presence of ginsenosides, including ginsenoside-Rb_1_, Rb_2_, Rc, Rd, Rg_1_, Re, Rf and Ro, which are also considered the critical biomarkers for the ginseng quality evaluation [5,6,7]. To date, ginseng has been well investigated in Western countries as a high reputation food supplement. Meanwhile, as a major region for ginseng cultivation, China has also approved a national project that in 2012 implemented ginseng cultivated for less than 5 years as a new food resource [8], which will further promote ginseng consumption and cultivation and finally make ginseng a table food for the broader population. In order to solve the current shortages of ginseng supply for commercial and scientific applications, scientific cultivation and effective usage of ginseng materials have attracted special attention for increasing the yield of ginseng.

As a member of the genus *Panax* and family Araliaceae, ginseng is an herbaceous perennial plant and has an incredible lifespan of hundreds of years. Owing to its long life cycle and significantly tonic effect, ginseng has been publicly recognized as “the king of all medical herbs” in China. As we all know, ginseng grows very slowly and needs at least 5 to 6 years cultivation to be used in the medical clinic, indicating that for ginseng the duration of cultivation is an important factor for evaluating its quality. Thus it is widely accepted that the longer the time ginseng grows, the better quality it has, which has been proved by many previous studies reporting how the amounts of total accumulated ginsenosides increase continuously with the duration of cultivation [9,10,11]. Although great efforts have been devoted to study the variations of ginsenoside content of ginseng of different cultivation regions, age, environment and harvest time [12,13,14,15,16], data on the accumulation and amounts of change of ginsenoside levels in different tissues cultivated for longer periods are still missing.

Actually, the saponins in ginseng have similar chemical structures, and they always present the synergistic effect in curing various diseases [17,18]. However, their pharmacological effects are also different and even opposite in some biological measurements [19,20,21]. For example, ginsenoside Rc and Rb_1_ of the protopanaxadiol (PPD) type inhibit AAPH-induced hemolysis in a concentration-dependent manner, while Rg_1_ and Rh_1_ of the protopanaxatriol (PPT) type improve the hemolysis with increasing concentration [22]. Since the pharmacological action of ginseng is not just the simple sum of its various components, the determination of the exact components and the amount of various ginsenosides in different tissues is crucial for determining the pharmacological efficacy of ginseng. Thus it is necessary to focus on the compositional variation among ginsenosides when evaluating the quality of ginseng besides year of cultivation. Furthermore, many medicinal parts of *Panax* ginseng such as root, rhizome and leaf are used in the clinic. The variations of ginsengosides among different ginseng tissues, especially for the root and rhizome, may lead to significant differences in clinical efficacy. Morphologically, the root of *Panax* ginseng has a long twisted rhizome called “Lutou” in Traditional Chinese Medicine (TCM). According to TCM experience, the root of ginseng is used in the clinic after removing “Lutou” due to its emetic effect. However, at present a great number of studies have proven that “Lutou” does not possess any perceived vomit-causing effect [23,24], and there is no significant difference in bioactivities between “Lutou” and root [25,26]. The use in the clinic of ginseng root together with “Lutou” began to be recorded in the 2005 version of the China Pharmacopoeia. However, the comparison of ginsenoside contents and composition between the root and rhizome has still not been systematically studied. Meanwhile, ginseng leaf is rich in ginsenosides and has been treated as a new crude drug in the 2005 version of the China Pharmacopoeia [27], but the clinical applications of ginseng leaf are different from those of the ginseng root. In addition, ginseng leaf is an ideal ingredient added into skin care products due to its strong antioxidation properties, whereas its medical effects for skin care still need further investigation. However, unlike the root and rhizome, the ginseng leaves can be harvested every year, and the variety of ginsenosides and antioxidation effects of different aged ginseng leaves is still unknown. Therefore, it is essential to investigate those changes of ginseng leaves from different years of cultivation, which will provide a scientific basis for the full usage of ginseng resources. To our best knowledge, many analytical approaches have been developed to identify and analyze ginsenosides from ginseng, and so far more than 40 ginsenosides have been identified [2]. Among these methods, high-performance liquid chromatography (HPLC) coupled with ultraviolet (UV) detection is the most commonly used method for the quantification of ginsenosides from ginseng [28,29]. However, as compared with ultra- performance liquid chromatography (UPLC) analytical methods, the HPLC-UV method shows some shortcomings such as being time-consuming (>60 min), its low sensitivity of the tested signal and so on. UPLC is a more powerful tool to simultaneously analyze multiple components in crude drugs with high speed and sensitivity, especially for measuring a large deal of analytical samples [30,31,32].

In this study, we established a UPLC-UV-MS method to identify and measure eight ginsenosides from ginseng within 13 min. Through using the developed method, the characteristic components in the leaf, rhizome and main root of ginseng were identified. Meanwhile, the contents of the eight main ginsenosides in the three tissues aged from 1 to 13 years were compared. The distributions of these components in the inner tissues of main root were profiled. Meanwhile, the antioxidant capacities of ginseng leaves from different aged ginseng were also investigated using the DPPH, ABTS and HRSA assays, respectively.

## 2. Results and Discussion

### 2.1. Identification of Ginsenosides and Quantitative Method Validation

**Figure 1:**
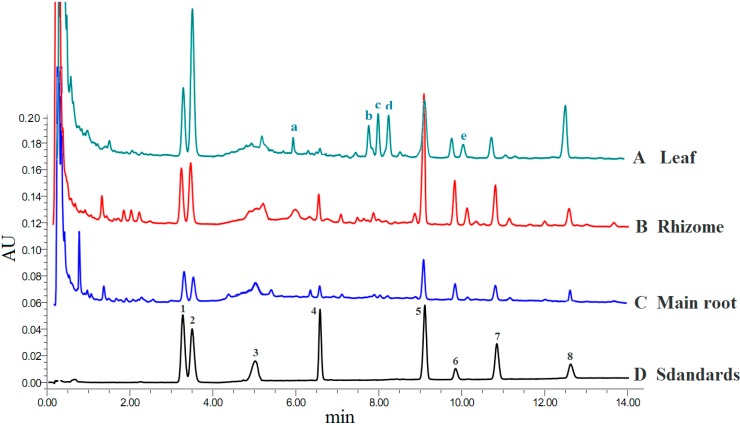
UPLC ginsenoside profile of *P*. *ginseng*: (A) Leaf; (B) Rhizome; (C) Main root; (D) Standards. Detection at 203 nm wavelength. Peaks: (1) ginsenoside-Rg_1_; (2) ginsenoside-Re; (3) ginsenoside-Ro; (4) ginsenoside-Rf; (5) ginsenoside-Rb_1_; (6) ginsenoside-Rc; (7) ginsenoside-Rb_2_ and (8) ginsenoside-Rd; (a) ginsenoside Ma-ginsenoside Re; (b) ginsenoside Rg_2_; (c) ginsenoside F_5_; (d) ginsenoside F_3_; (e) ginsenoside Rb_3_.

UPLC-MS analysis was performed using a Waters ACQUITY UPLC system, and the markers and characteristic peaks were identified according to their fragment ions (*m/z*) and retention time. Figure 1 shows the UPLC profiles of leaf, rhizome and main root of 12 year-old ginseng, in which leaf and rhizome extracts show more characteristic peaks than main root extract.

**Figure 2:**
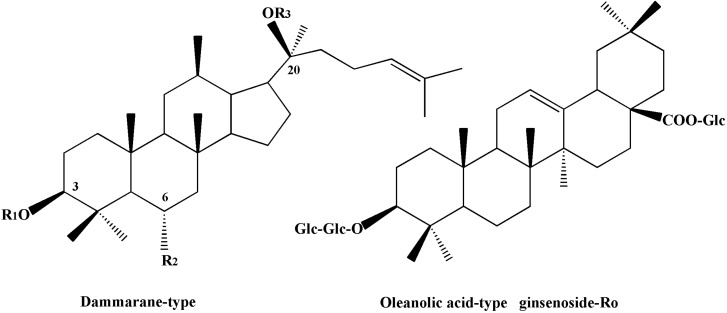
Chemical structures of eight ginsenosides.

The main characteristic peaks a-e were identified as Ma-ginsenoside Re (*m/z*: 1,031, 1,007, 1,024); ginsenoside Rg_2_ (*m/z*: 829, 846, 897); ginsenoside F_5_ (*m/z*: 815, 832); ginsenoside F_3_ (*m/z*: 815, 832) and ginsenoside Rb_3_ (MS: 1,123, 1,163, 1,231), respectively. The fragment ions all showed in the modes of [M+2Na]^+^ or [M+2Na−2H]^+^. An external standard was utilized in order to the quantitative determination of ginsenosides. All tested ginsenosides belong to the dammarane type except for the single example of an oleanolic acid-type in Ro, and their structures are listed in Figure 2.

**Table:** 

Compounds	R_1_	R_2_	R_3_
Ginsenoside-Rb1	Glc(2-1) Glc	H	Glc(6-1) Glc
Ginsenoside-Rb2	Glc(2-1) Glc	H	Glc(6-1) Arap
Ginsenoside-Rc	Glc(2-1) Glc	H	Glc(6-1) Araf
Ginsenoside-Rd	Glc(2-1) Glc	H	Glc
Ginsenoside-Re	H	O- Glc(2-1) Rha	Glc
Ginsenoside-Rf	H	O- Glc(2-1) Glc	H
Ginsenoside-Rg1	H	O-Glc	Glc

The calibration curves were plotted with a series of concentrations of standard solutions. Each marker curve was measured at a minimum of six levels. The promising linear correlations and high sensitivity at these conditions were confirmed by the correlation coefficients (r^2^, 0.9990–0.9994). Limits of detection (LOD) and limits of quantification (LOQ) were also acquired by testing noise-signal ratios, respectively. The detailed regression equation and validation data of each marker are listed in Table 1.

**Table 1:** Regression data, linear ranges, LODs and LOQs for the eight markers of the assay.

Markers	Regression Equation	R^2^	Linear Range (μg/mL)	LOD (μg/mL)	LOQ (μg/mL)
Ginsenoside Rg_1_	*y* = 785.09 *x* − 49305	0.9991	49.73–1823.33	2.10	5.21
Ginsenoside Re	*y* = 1015.2 *x* − 86311	0.9990	39.73–910.42	2.19	4.86
Ginsenoside Ro	*y* = 1256.8 *x* − 77168	0.9993	29.77–873.33	1.31	3.44
Ginsenoside Rf	*y* = 1262.7 *x* − 80147	0.9994	25.86–948.33	1.72	4.09
Ginsenoside Rb_1_	*y* = 912.66 *x* − 85373	0.9993	47.73–1750.00	0.98	4.41
Ginsenoside Rc	*y* = 577.06 *x* − 13760	0.9993	30.53–447.78	1.01	4.53
Ginsenoside Rb_2_	*y* = 992.69 *x* − 61958	0.9993	24.86–911.67	1.82	3.79
Ginsenoside Rd	*y* = 991.01 *x* − 22351	0.9992	28.11–412.22	1.63	4.63

It can be seen from Figure 1 that the characteristic peaks (a–e) are mainly distributed in the leaf and rhizome, while they are hardly detected in the main root. The contents of ginsenosides corresponding to peaks b, c, d calculated by peak area are higher in the leaf extract than those in the rhizome and main root, indicating that the ginsenosides corresponding to ginsenoside Rg_2_, ginsenoside F_5_ and ginsenoside F_3_ are the characteristic components in the leaf.

The eight detected constituents have good resolution and can be easily determined and they are also the main constituents in the assayed samples. The established UPLC-UV-MS quantitative method has the good linear ranges and accords to the relative requirement.

### 2.2. Ginsenosides Contents in Different Tissues of the Same Ginseng Plant

Under the same chromatographic conditions used for the markers, all samples were analyzed in triplicate. The identification and qualitative detection was conducted based on the MS information and retention time of each standard, and the calibrations were used to quantify the contents of biomarkers in the tested samples. Three different tissues corresponding to leaf, rhizome and main root subjected to different growing durations were selected to analyze the contents of eight bioactive compounds. Through comprehensive analysis of the data in Table 2, the contents of all eight detected ingredients in leaf extracts have the highest levels as compared with those in the rhizome and main root within the same cultivation duration. The total contents of ginsenosides reach 30 mg/g in leaf extract from 1 year-old ginseng, while the rhizome and main root need at least eight years of growth to accumulate the same level of ginsenosides as the leaves. Although the amounts of ginsenosides accumulated in the three detected tissues represent the different growth rates, their overall levels all increase with years of cultivation. The ginsenosides of leaf extract have a relatively slower rate of increase within a 13 year growth cycle as compared with those of other two tissues. The contents of ginsenosides in the leaf samples vary from 36.64 to 59.03 mg/g, which indicates an approximate doubling in the ginseng samples grown from 1 to 13 years. However, the data have some contradictions with a previous report that the total content of ginsenosides in ginseng leaf decreases with increasing age [33]. The inconsistent results may be caused by the harvest time, different species and areas of cultivation, which are considered as the important factors for determining the quality of ginseng and affect the accumulated amounts of ginsenosides.

Compared with the amounts of ginsenosides accumulated in leaf samples, the amounts in the rhizome samples show a downward trend after a first rise, followed by values varying from 10.76 to 65.30 mg/g within the studied 13 year growth cycle. In addition, it is worth noting that the amounts of ginsenosides accumulated in rhizome samples increased sharply from 24.48 to 43.92 mg/g during the 7th and 8th growth year. Among these three detected tissues, the main root has the lowest contents of ginsenosides in 1 to 13 year-old ginseng, with a range from 16.27 to 31.14 mg/g. Furthermore, the pattern of ginsenoside increases in the main root samples presents a rapid-growth period from 16.27 to 23.48 mg/g in the first four growth years, and then remains a stable level of about 24 mg/g until the eighth growth year, subsequently following by a fast-growth time up to 13 years of age. The amounts of eight active ingredients accumulated in the main root also follow a similar growth trend as those in leaf and rhizome extracts. Meanwhile, through analyzing the data in Table 2, ginsenoside Rg_1_, Re and Rb_1_ are the major constituents in main root and rhizome. By contrast, the content of ginsenoside Rb_1_ is relatively low in leaf extracts, while ginsenoside Rg_1_ and Re represent the dominant components, indicating that the contents and compositions of ginsenosides in the three tissues differ from each other. The year of cultivation is thus an important factor affecting the amounts of the active components accumulated in these three tested ginseng tissues.

**Table 2:** Contents of eight ginsenosides from different tissues of *panax ginseng* (mg/g).

Samples	Rg_1_	Re	Ro	Rf	Rb_1_	Rc	Rb_2_	Rd	Total	PPT-/PPD-Type
**Leaves**										
1L	6.43 ± 0.12	10.45 ± 0.32	2.93 ± 0.32	2.63 ± 0.23	4.41 ± 0.08	2.26 ± 0.12	3.29 ± 0.05	4.26 ± 0.33	36.64 ± 0.56	1.37
2L	5.98 ± 0.04	8.76 ± 0.14	2.79 ± 0.11	2.58 ± 0.21	3.88 ± 0.04	1.31 ± 0.25	2.73 ± 0.08	2.24 ± 0.34	30.27 ± 0.73	1.71
3L	10.84 ± 0.10	16.34 ± 0.22	3.91 ± 0.43	2.68 ± 0.53	4.23 ± 0.42	1.53 ± 0.27	2.82 ± 0.13	2.43 ± 0.14	44.79 ± 1.29	2.71
4L	14.70 ± 0.08	11.05 ± 0.31	4.14 ± 0.04	2.76 ± 0.08	4.06 ± 0.16	1.36 ± 0.16	2.77 ± 0.54	1.89 ± 0.10	42.74 ± 0.38	2.83
5L	12.72 ± 0.26	20.18 ± 0.11	4.27 ± 0.56	2.68 ± 0.16	3.82 ± 0.02	1.79 ± 0.17	2.86 ± 0.20	2.87 ± 0.07	51.20 ± 1.10	3.14
6L	11.03 ± 0.13	17.04 ± 0.07	4.18 ± 0.19	2.69 ± 0.32	3.90 ± 0.18	1.95 ± 0.33	3.17 ± 0.43	4.07 ± 0.22	48.04 ± 0.37	2.35
7L	13.55 ± 0.07	15.27 ± 0.08	4.38 ± 0.42	2.74 ± 0.67	4.40 ± 0.38	2.19 ± 0.26	3.24 ± 0.11	3.75 ± 0.14	49.51 ± 1.15	2.32
8L	9.59 ± 0.05	17.35 ± 0.43	5.00 ± 0.34	2.65 ± 0.32	4.15 ± 0.05	1.54 ± 0.05	2.82 ± 0.03	2.12 ± 0.55	45.22 ± 0.91	2.78
9L	9.15 ± 0.08	17.11 ± 0.32	4.00 ± 0.09	2.74 ± 0.05	4.07 ± 0.25	1.51 ± 0.15	3.08 ± 0.25	2.10 ± 0.38	43.76 ± 0.39	2.70
10L	13.24 ± 0.11	18.47 ± 0.29	4.63 ± 0.86	2.98 ± 0.19	4.11 ± 0.34	2.82 ± 0.10	3.52 ± 0.21	3.57 ± 0.29	53.34 ± 0.83	2.48
11L	9.09 ± 0.21	17.01 ± 0.55	4.42 ± 0.57	2.75 ± 0.24	3.91 ± 0.41	1.62 ± 0.42	3.03 ± 0.10	2.77 ± 0.20	44.59 ± 0.30	2.55
12L	10.81 ± 0.20	18.46 ± 0.37	4.20 ± 0.27	2.69 ± 0.15	5.16 ± 0.16	3.78 ± 0.37	4.46 ± 0,18	6.49 ± 0.17	56.06 ± 1.42	1.61
13L	13.69 ± 0.34	20.87 ± 0.22	4.28 ± 0.09	2.69 ± 0.27	4.08 ± 0.06	3.10 ± 0.26	3.89 ± 0.06	6.44 ± 0.11	59.03 ± 0.63	2.13
**Rhizome**										
1R	2.57 ± 0.06	3.58 ± 0.30	ND	ND	ND	1.10 ± 0.06	2.55 ± 0.26	0.96 ± 0.02	10.76 ± 1.06	1.33
2R	5.06 ± 0.10	5.55 ± 0.14	5.41 ± 0.12	2.58 ± 0.11	5.45 ± 0.10	3.16 ± 021	3.21 ± 0.52	1.46 ± 0.17	31.88 ± 0.89	0.99
3R	3.40 ± 0.33	5.01 ± 0.19	2.63 ± 0.15	2.76 ± 0.10	5.16 ± 0.42	2.28 ± 0.17	3.33 ± 0.16	1.61 ± 0.21	26.19 ± 0.63	0.90
4R	4.13 ± 0.26	4.68 ± 0.09	2.60 ± 0.48	2.97 ± 0.09	5.20 ± 0.69	2.25 ± 0.09	3.15 ± 0.25	1.46 ± 0.13	26.45 ± 0.73	0.98
5R	3.66 ± 0.18	4.77 ± 0.31	2.69 ± 0.38	2.77 ± 0.39	5.24 ± 0.19	2.30 ± 0.37	3.35 ± 0.10	1.37 ± 0.19	26.15 ± 1.29	0.91
6R	3.37 ± 0.14	4.23 ± 0.40	2.65 ± 0.35	2.69 ± 0.47	4.55 ± 0.05	1.59 ± 0.15	2.88 ± 0.18	1.13 ± 0.30	23.09 ± 0.30	1.01
7R	3.54 ± 0.12	4.30 ± 0.59	2.59 ± 0.14	2.82 ± 0.35	5.10 ± 0.21	1.83 ± 0.28	3.00 ± 0.14	1.30 ± 0.28	24.48 ± 0.47	0.95
8R	5.78 ± 0.30	8.29 ± 0.65	3.35 ± 0.14	3.29 ± 0.19	9.62 ± 0.09	5.71 ± 0.32	5.14 ± 0.08	2.75 ± 0.35	43.92 ± 1.28	0.75
9R	5.96 ± 0.72	7.00 ± 0.21	3.25 ± 0.19	3.28 ± 0.18	9.96 ± 0.49	4.54 ± 0.07	4.72 ± 0.26	2.12 ± 0.14	40.83 ± 0.14	0.76
10R	5.55 ± 0.36	6.96 ± 0.45	3.27 ± 0.05	3.49 ± 0.19	8.57 ± 0.22	5.05 ± 0.14	4.77 ± 0.14	2.14 ± 0.16	39.79 ± 0.51	0.78
11R	5.85 ± 0.44	6.69 ± 0.33	3.30 ± 0.27	3.49 ± 0.19	9.49 ± 0.10	4.45 ± 0.26	4.53 ± 0.39	1.77 ± 0.23	39.56 ± 0.76	0.79
12R	9.23 ± 0.07	9.42 ± 0.16	3.86 ± 0.23	4.24 ± 0.18	15.36 ± 0.20	7.53 ± 0.29	6.30 ± 0.52	2.78 ± 0.39	58.73 ± 1.37	0.72
13R	11.95 ± 0.22	10.26 ± 0.10	4.19 ± 0.11	4.27 ± 0.12	16.43 ± 0.29	8.43 ± 0.47	7.04 ± 0.82	2.74 ± 0.29	65.30 ± 0.11	0.76
**Main root**										
1 MR	2.81 ± 0.17	4.16 ± 0.50	ND	ND	4.06 ± 0.26	1.34 ± 0.31	2.73 ± 0.26	1.17 ± 0.18	16.27 ± 0.57	0.75
2 MR	2.84 ± 0.63	4.32 ± 0.24	ND	2.63 ± 0.53	4.26 ± 0.17	1.57 ± 0.30	2.8 ± 0.10	1.38 ± 0.09	19.79 ± 1.25	0.98
3 MR	3.1 ± 0.30	4.39 ± 0.25	ND	2.68 ± 0.10	4.45 ± 0.18	1.91 ± 0.19	3.01 ± 0.16	1.23 ± 0.12	20.77 ± 0.41	0.96
4 MR	3.77 ± 0.84	4.22 ± 0.19	2.51 ± 0.42	2.84 ± 0.37	4.51 ± 0.17	1.67 ± 0.13	2.76 ± 0.21	1.19 ± 0.28	23.48 ± 0.66	1.07
5 MR	3.56 ± 0.12	4.43 ± 0.10	2.53 ± 0.37	2.78 ± 0.30	4.75 ± 0.20	2.03 ± 0.52	3.18 ± 0.28	0.90 ± 0.28	24.17 ± 0.53	0.99
6 MR	3.5 ± 0.18	4.68 ± 0.37	2.53 ± 0.10	2.78 ± 0.72	4.89 ± 0.20	2.03 ± 0.22	3.06 ± 0.14	1.21 ± 0.21	24.68 ± 1.40	0.98
7 MR	3.6 ± 0.24	4.44 ± 0.17	2.53 ± 0.19	2.79 ± 0.31	5.19 ± 0.28	1.82 ± 0.28	2.89 ± 0.10	1.08 ± 0.30	24.34 ± 0.39	0.99
8 MR	3.48 ± 0.27	4.68 ± 0.06	2.51 ± 0.25	2.79 ± 0.19	5.23 ± 0.08	1.87 ± 0.14	2.9 ± 0.02	1.2 ± 0.17	24.68 ± 1.44	0.98
9 MR	4.74 ± 0.38	5.39 ± 0.42	3.46 ± 0.19	2.97 ± 0.08	6.54 ± 0.04	2.55 ± 0.17	3.51 ± 0.11	1.1 ± 0.41	30.26 ± 0.18	0.96
10 MR	4.48 ± 0.47	4.89 ± 0.11	3.49 ± 0.04	2.98 ± 0.59	5.59 ± 0.63	2.44 ± 0.21	3.34 ± 0.08	1.17 ± 0.60	28.39 ± 1.30	0.98
11 MR	5.8 ± 0.26	5.21 ± 0.05	3.92 ± 0.51	3.13 ± 0.41	7.00 ± 0.37	2.72 ± 0.41	3.61 ± 0.13	1.16 ± 0.53	32.55 ± 0.77	0.98
12 MR	5.98 ± 0.08	5.21 ± 0.15	3.79 ± 0.18	3.00 ± 0.36	4.8 ± 0.42	3.12 ± 0.15	2.79 ± 0.26	1.14 ± 0.59	29.83 ± 1.57	1.2
13 MR	6.16 ± 0.25	5.77 ± 0.26	3.19 ± 0.10	3.26 ± 0.32	4.43 ± 0.27	3.23 ± 0.10	3.84 ± 0.51	1.26 ± 0.10	31.14 ± 0.62	1.19

### 2.3. Variation of Ginsenoside Heterogeneity in Different Tissues

In addition to the change of total content of ginsenosides in the different tissues, the ratio of two dammarane types of ginsenosides also differs in different tissues as the years of cultivation increase. Structurally, dammarane-ginsenosides are classified into protopanaxadiol (PPD) and protopanaxatriol (PPT) types, according to the different positions of aglycones linked to the parent nucleus structure. As shown in Table 2, the proportions of PPT type (Rg_1_, Re and Rf) to PPD type (Rb_1_, Rb_2_, Rc and Rd) in the leaf samples are the highest among the three analyzed tissues, which are larger than 2.00 on average and not obviously dependent on the duration of cultivation. The PPT-/PPD-type radios in the leaf samples present first a rising trend and then descend and the trend is repeated with different rates. Compared with the irregular change of the proportions in leaf extracts, the PPT-/PPD-type ratios in the rhizome exhibit a declining Z-type tendency, ranging from 1.33 to 0.76 with a sharp change between the seventh and eighth growth year. The ratio curve in the rhizome is divided into two stages without any transition phase, the first stable period seen in 2 to 7 year-old samples ranged from 0.90~1.01 and the second period ranged from 0.72~0.79 in the following six years, while the proportions of the PPT-/PPD-type in the main root samples are close to 1.00 from 2 to 11 growth years, and then rise to 1.20 in the last two growth years. Through the above analysis, it can be concluded that the variations of the PPT-/PPD-type ratio in different tested tissues are diverse and may result in alteration of relative biological activities. The alteration of the proportions of the two dammarane types of ginsenosides may attribute to the inconsistent increases in the amounts of individual ingredients seen along with years of cultivation.

**Figure 3:**
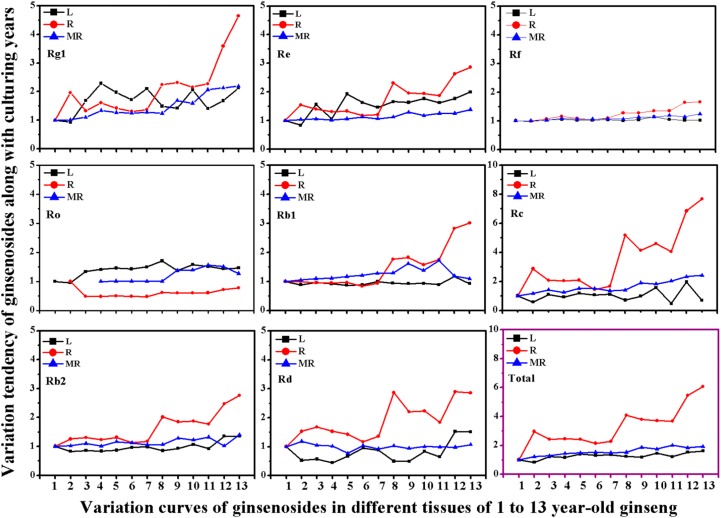
The variation ratios of eight individual ginsenoside in leaf (L), rhizome (R) and main root (MR) of 1 to 13 year-old ginseng. Values show mean ± SE of three experiments performed in triplicate. (The defect points mean the components are not detected).

In order to reveal the cause of the changes in PPT-/PPD-type proportion, the ratios of change of single ginsenosides in different tissues should be evaluated along with years of cultivation. As seen in Figure 3, the content of eight ginsenosides at 1 year of age is taken as the basic accumulation amount, which is used as the standard to compare with that of other growth periods. The variance of the ratio of individual ginsenosides also occurs in an organ-specific manner corresponding to the order rhizome > main root > leaf. Among the eight ginsenosides, ginsenoside-Rc displays the highest ratio change of up to 7.66 from 1 to 13 year-old samples in the rhizome, followed by ginsenoside-Rg_1_ and Rb_1_ changes to 4.65 and 3.01, respectively. Similar to the results seen in the rhizome, ginsenoside-Rc and Rg_1_ also show relatively higher changing ratios of up to 2.41 and 2.19 in the main root. Moreover, the ratios of change of the ingredients in main root rise steadily. By contrast with the main root and rhizome, the eight components in the leaf samples have the lowest growth ratios and change erratically overall, except for ginsenoside-Re and Ro. The relatively stable curves of Rf and Ro indicate that the amounts of the two components accumulated in all three tissues vary slightly along with duration of cultivation. The change ratios have a similar and smooth rise for total ginsenosides in the main root and leaf extracts, while it rises substantially in the rhizome samples with aging. Therefore, the different rates of change of each single ginsenoside leads to the observed variances of ginsenoside heterogeneity, subsequently resulting in possibly different biological activities in different ginseng tissues.

In addition, Shan *et al.* [10] have verified the close association between bioactivity and year of cultivation of ginseng, and the different contents and proportion of ginsenosides are the major reason for the variation in the bioactivity of ginseng. Besides the contents of ginsenosides, the compositions of active components are very important for the bioactivity of ginseng. The two types of ginsenosides play different roles in some bioactivities, and their compositional differences among ginsenosides are a key factor for this [34,35,36]. The optimal combination may provide the strongest bioactivities, that is, the best composition-activity relationship. Therefore, the changes of PPT-/PPD-type ratio will result in variance of the potency of bioactivities and further alter the overall quality of ginseng. In the present study, the PPT-/PPD-type ratio in the main root samples shows a very stable relationship with the duration of cultivation, which can effectively ensure the clinical quality of ginseng root, while the other two parts exhibit converse tendencies in the ratio, resulting in possible variances of pharmacological activities.

### 2.4. Ginsenoside Distribution Patterns in Four Parts of the Main Root

**Figure 4:**
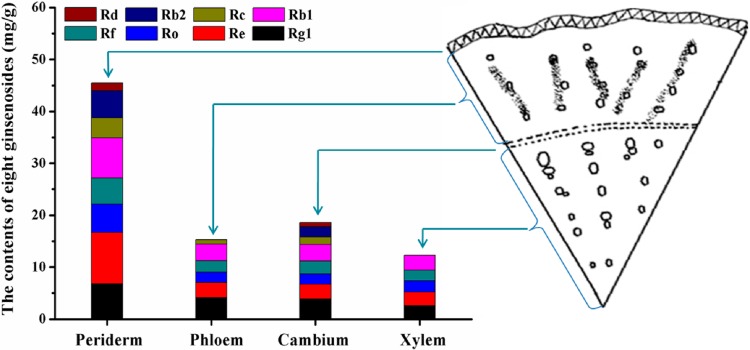
The distribution of eight ginsenosides in the periderm, phloem, cambium, and xylem of 5 year aged ginseng.

As the major medicinal part, the main root consists of four different inner parts, including periderm, phloem, cambium and xylem. Ginsenoside accumulations in inner parts of the main root aged 5 years also showed a specific enrichment pattern. As shown in Figure 4, compared with other inner parts of the cross section of main root, the outmost periderm has the highest enrichment of ginsenosides, which was 2.97, 2.45 and 3.70 times that in phloem, cambium and xylem, respectively.

The peaks corresponding to the eight identified ginsenosides are found in the inner parts of the periderm and cambium, while ginsenoside-Rb_2_, Rd and Rc cannot be detected in the xylem part. In addition to ginsenoside Rb_2_, ginsenoside Rd is also absent in phloem part. As for the proportions of the analyzed ginenosides, ginsenoside-Rg_1_, Re and Rb_1_ are the main components in inner parts of the main root, which is in good agreement with previous studies [37]. It can be concluded that ginsenosides in the inner parts of the main root are mainly located in the periderm of roots, which is also consistent with histochemical findings [38]. At the meantime, it also provides us valuable information about the best way of processing ginseng products, that is, the quality of ginseng products would be enhanced by not peeling the periderm.

### 2.5. Antioxidant Evaluation of Ginseng Leaves

It is well known that ginseng has a positive anti-aging effect. Meanwhile, according to the previous relative studies, the radical-induced oxidations of lipids and membranes have been proven to be major contributors to the chemopathogenesis of aging [39,40]. Thus many efforts have focused on investigating the antioxidant activity of ginseng, in a bid to understand the mechanism of action of the antioxidation effects of ginseng which help avoid various diseases resulting from free radical oxidation.

Ginseng leaf is a more easily available and lower valuable resource compared with the root and rhizome parts of ginseng, and it has also been proven to possess antioxidant properties [41]. Jung *et al.* [42] revealed that the antioxidant properties presented a discrepancy between cultivated and wild ginseng leaves. As the leaf of ginseng can be harvested each year, whether its antioxidant activities vary following the trend of the contents of ginsenosides with year of cultivation is still unclear. Therefore, it is necessary to investigate the change of the antioxidant potencies in ginseng leaf with different growth duration, which will provide a great deal of useful information to further promote the full application of ginseng leaves.

The antioxidant activities of leaf samples from 1 to 13 year-old ginseng were tested. In order to obtain a comprehensive evaluation about the antioxidant effect, three different *in vitro* radical scavenging assays were applied, including scavenging capacity against 2,2-diphenyl-1-picrylhydrazyl radical (DPPH), 2,2'-azinobis-(3-ethylbenzthiazoline-6-sulphonate), free radical-scavenging capacity (ABTS) and hydroxyl radical-scavenging activity (HRSA). Table 3 shows the antioxidant properties of leaf extracts from ginseng samples over 13 years of cultivation. The results are presented as Trolox equivalents (TE). It can be clearly observed that all the leaf samples possess scavenging effects in these three assays, and the capacities show an increasing tendency with the duration of cultivation. The antioxidant activities in the leaf samples from 1 to 13 year-old ginseng are significantly increased by 49.20%, 45.56% and 55.83% in the DPPH, ABTS and HRSA assay, respectively. The higher the rate of DPPH/ABTS/HRSA consumption obtained, the more powerful the antioxidant capacity a sample possesses, indicating that the highest activities are presented by the leaf extracts from the 10-year old ginseng that reaches 48.40 ± 3.61, 74.26 ± 0.34 and 31.26 ± 0.68 μg TE/mg, respectively. The antioxidant activities of leaf extracts from different aged ginseng samples were explored using three different measurement methods. Through analyzing the results of these three measurements, the DPPH results are agreement with the ABTS values and correlated with the HRSA results as well, as polyphenols and flavonoids in plants are always believed to be natural antioxidants that can effectively scavenge free radicals [43,44]. In the meanwhile, previous research has also revealed the close correlation between these constituents and the antioxidant activities in ginseng leaf. Herein, it is interesting to see that the contents of ginsenosides and antioxidant capacities all present a similarly rising trend. By utilizing the SPSS 17.0 software, the correlation coefficient is calculated to be larger than 0.91 (*p* < 0.05), suggesting that the antioxidant activities have a strong correlation with the amounts of accumulated ginsenosides. The antioxidant activity results reveal that the leaf samples from samples grown longer exhibit higher antioxidant activities, resulting from the increased accumulation of ginsenosides.

In conclusion, the results show a notable antioxidant activity in all leaf extracts, and a strong correlation between antioxidant capacities and the contents of ginsenosides. The highest antioxidant activity is tested for the leaf extracts from the 10-year old ginseng sample.

**Table 3:** Antioxidant capacity of ginseng leaf extracts from 1–13 year-old ginseng samples.

Samples	DPPH (μg TE/mg)	ABTS (μg TE/mg)	HRSA (μg TE/mg)
**1**	33.75 ± 1.57	52.45 ± 1.39	21. 87 ± 0.67
**2**	32.44 ± 2.32	51.02 ± 2.17	20.06 ± 0.34
**3**	38.73 ± 1.08	56.93 ± 0.90	23.52 ± 0.81
**4**	36.26 ± 0.79	57.45 ± 2.86	23.72 ± 1.45
**5**	46.10 ± 1.49	72.27 ± 0.52	29.73 ± 2.13
**6**	46.08 ± 0.21	71.08 ± 0.49	28.17 ± 0.87
**7**	46.36 ± 0.46	71.66 ± 2.25	28.66 ± 0.66
**8**	42.38 ± 1.15	66.38 ± 0.29	25.88 ± 0.62
**9**	41.12 ± 0.62	62.65 ± 1.02	25.08 ± 2.14
**10**	48.40 ± 3.61	74.26 ± 0.34	31.26 ± 0.68
**11**	41.82 ± 0.84	65.92 ± 1.54	25.49 ± 1.46
**12**	43.30 ± 0.05	71.30 ± 0.55	27.90 ± 0.82
**13**	41.06 ± 2.91	66.36 ± 1.01	25.96 ± 0.55

Notes: Results are mean ± standard deviation of three independent measurements; TE: Trolox equivalents.

## 3. Experimental Section

### 3.1. Plant Materials

The fresh *P. ginseng* samples aged from 1 to 13 years, belonging to the Shizhu ginseng cultivar (similar to wild ginseng), were collected from Shizhu Village in Liaoning Province, China, in August 2013. The samples were taxonomically identified by the Institute of Special Animal and Plant Science, Chinese Academy of Agricultural Sciences. All samples consisted of five individuals except the Shizhu 11–13 year samples with three individuals.

### 3.2. Reagents and Standards

HPLC grade acetonitrile and methanol were purchased from Thermo Fisher Scientific (Norcross, GA, USA); Ultra-pure water was prepared on a Milli-Q water purification system (Millipore, Billerica, MA, USA). The standards of ginsenosides-Rg_1_, Re, Rf, Rb_1_, Rb_2_, Rc and Rd were purchased from the National Institute for the Control of Pharmaceutical and Biological Products (Beijing, China), while the ginsenoside Ro standard was obtained from Shanghai Winherb Medical Technology Co., Ltd (Shanghai, China). In order to guarantee the UPLC analysis requirements, the purity of all standards was more than 98%. 2,2'-Azinobis-3-ethylbenzotiazoline-6-sulphonic acid (ABTS) and 2,2-diphenyl-1-picrylhydrazyl (DPPH) were purchased from Sigma-Aldrich (Steinheim, Germany); All other reagents were of analytical grade (all over 97% purity) and purchased from the Beijing Chemical Factory (Beijing, China).

### 3.3. Sample and Standard Solutions Preparation

The whole fresh plant was thoroughly rinsed with deionized water, and then cut into three different parts including leaves, rhizome and main root. The four parts in the cross section of 5 year-old main root, namely the xylem, cambium, phloem and periderm, were peeled off to detect the changes in ginsenosides of these parts, which can clearly exhibit the distribution patterns of ginsenosides in the internal tissues. Then all the cut samples were dried in an oven at 50 °C for 48 h, and finally smashed into a powder.

Approximately, 1.0 g of pulverized sample was weighed out and extracted according to the pharmacopeia method with slight modification [45]. Subsequently, the powder sample was wrapped with a filter paper and then put into a Soxhlet extractor to reflux with 80 mL chloroform (CHCl_3_) at 60 °C for 3 h. After the refluxing process, the treated sample was dried in the fume hood and put in a 100 mL flask with 50 mL *n*-butyl alcohol. After soaking overnight at room temperature (about 12 h), the solution was ultrasonically extracted for 1 h and finally the volume was adjusted to 50 mL. After evaporation at 55 °C, the residue was dissolved in 4 mL of 100% methanol, and then filtered through a 0.22 μm nylon membrane filter for UPLC analysis. All samples were prepared according to the above mentioned extraction method.

In order to establish the calibration curves of eight standards (ginsenosides-Rg_1_, Re, Rf, Rb_1_, Rb_2_, Rc, Rd and Ro), a stock solution containing the eight standards was precisely weighed and dissolved in methanol, and then these solutions were diluted to a series of standard solutions with gradient concentrations. Meanwhile, in order to identify the retention time of individual ingredients, solutions of each standard were also prepared separately. All solutions were stored in a refrigerator at 4 °C for analysis.

### 3.4. Apparatus and UPLC Analytical Conditions

The prepared samples were analyzed by using a Waters ACQUITY UPLC system (Waters, Milford, MA, USA) coupled with a Q-Tof mass spectrometer and a photodiode array detector (PDA). The experimental data was collected and processed on an Empower 3.0 chromatographic work station. All samples of ginseng were separated on a Waters ACQUITY UPLC BEH C_18_ (2.1 × 50 mm, 1.7 μm) column. The mobile phases were water (solvent A) and acetonitrile (solvent B) with gradient elution as follows: 0–4.0 min, 19% B; 4.0–7.0 min, 19%–30% B; 7.0–15.0 min, 30%–32% B; 15.0–16.0 min, 32%–80% B. Elution was performed at a flow rate of 0.50 mL/min. The column temperature was set to 25 °C. UV measurements were obtained at 203 nm and the injection volume for analysis was 2 μL.

### 3.5. In Vitro Antioxidant Activity Assays

The antioxidant capacities of the prepared leaf samples were measured by determining the free radical scavenging effect on 1,1-diphenyl-2- picrylhydrazyl (DPPH) radical according to the method described by Lee with slight modification [46,47]. Prior to analysis, a stock solution of DPPH with 39.4 mg·L^−1^ (100 μM) was freshly prepared by dissolving the reagent in 100% methanol. Five μL of the original extracts was added to 995 μL of DPPH solution, and then the solutions were mixed and left to completely react for 30 min at room temperature. The absorbance was measured at 517 nm wavelength using a UV-VIS Spectrophotometer equipped with a 96-cell holder (Varioskan Flash Multimode Reader, Thermo Fisher Scientific, Waltham, MA, USA) against a blank of methanol without DPPH. The percentage of DPPH consumption was converted to Trolox equivalents by using a calibration curve.

In the ABTS assay, according to the method described by Xu *et al.* [48], 7 mM ABTS solution and 2.45 mM potassium persulphate aqueous solutions were mixed and then left standing in the dark at room temperature for 24 h before being used. Five μL of the original extracts were added to 995 μL ABTS solution. The amount of ABTS radical consumed by the tested samples was measured at 735 nm wavelength after 20 min of reaction time. The percentage of ABTS consumption was transformed in Trolox equivalents by using a calibration curve.

HRSA was performed by using the method of Meng *et al.* [49]. Thus, 330 μL of FeSO_4_ (2 mM), 330 μL of salicylic acid (6 mM) and 10 μL of the original extracts were mixed together, and then 330 μL of H_2_O_2_ (1 mM) was added to the mixture solution to react for 20 min at 37 °C. The samples were measured at 593 nm wavelength. The results were also expressed as trolox equivalents.

### 3.6. Statistical Analysis

The experimental data was analyzed with one-way analysis of variance (ANOVA) by using SPSS 16.0. Duncan’s test at *p* < 0.05 was employed to compare the significant difference among means.

## 4. Conclusions

In this study, a slow age-dependent increase of ginsenosides is detected in the main root of *P. ginseng*. The amounts of accumulated ginseneosides increase fast in the first four years of growth, and then remain at a stable level during the following four years, and the fifth year of cultivation represents an important transition. The variance ratio of individual ginsenosides with age occurred in an organ-specific manner: rhizome > main root > leaf.

The patterns of ginsenoside accumulation in the root agree with the traditional view that ginseng grown for at least five years can be used in the clinic. In order to maximize economic profits and promote the quality of ginseng products at the same time, five years of growth is the optimal harvesting time for ginseng. Besides, the results also reflect from the ginsenoside contents that a better quality product can be obtained after eight years of growth comparing with that of 5 year-old plants. Though the amounts of ginsenosides accumulated in the rhizome and leaf parts have different growth rates, they both display similar change tendencies as the main root parts of ginseng. The accumulation of ginsenosides in the root periderm is 2-fold than the other parts of the cross-section of ginseng main root.

The results confirm the traditional medical views that more planting years can continuously enhance the quality of ginseng when using the content of ginsenosides as a marker. However, when considering the maximum economic benefit, ginseng should be grown for at least five years and the best harvest time is within 8 years. Besides, “Lutou” of less than 8 years of age can be used together with the root for their similar proportion of PPT/PPD. Furthermore, ginseng leaves have powerful antioxidation properties, which are closely correlated with the amounts of accumulated ginsenosides. This study provides a helpful basis for quality evaluation and expanding the promising commercial applications of ginseng products.
